# Supplementary material for: Geodesy of irregular small bodies via neural density fields
Source: Commun Eng. 2022 Dec 28;1:48. doi: 10.1038/s44172-022-00050-3 (PMC10956048; doi:10.1038/s44172-022-00050-3)
Supplement: Supplementary file 2 — Supplementary Information [file 44172_2022_50_MOESM2_ESM.pdf]

Supplementary Material for:

Geodesy of irregular small bodies via neural density fields: geodesyNets

Dario Izzo† and Pablo Gómez

Advanced Concepts Team, European Space Agency
European Space Research and Technology Centre (ESTEC)
Keplerlaan 1, 2201 AZ Noordwijk, The Netherlands

† Corresponding author. E-mail: dario.izzo@esa.int

Supplementary Methods

Supplementary Method 1

As loss function for a geodesyNet we investigated several standard machine learning losses such as the Mean Squared Error or the Mean Absolute Error. While such choices would already result in successful training, especially for regular body shapes, they can be considerably improved by assuming the network predictions $\hat{y}_i$ as biased by a factor κ. Then, given a batch of N ground-truth quantities $y_i, i = 1..n$ and predictions $\hat{y}_i, i = 1..n$, over a batch, the optimal value of κ can be found to be:

$$\kappa = \frac{\sum_{i=1}^n \hat{y}_i y_i}{\sum_{i=1}^n y_i^2}$$

This normalizing factor is further referred to as mass normalization factor and scales the neural model predictions so that the value $\kappa\hat{y}_i$ is used in the loss function. In the case of the Mean

Absolute Error, this produces the new loss function:

$$\mathcal{L}_{\kappa MAE} = \frac{1}{n}\sum_{i=1}^{n}|y_i - \kappa\hat{y}_i| = \frac{1}{n}\sum_{i=1}^{n}\left|y_i - \frac{\sum_{i=1}^{n}\hat{y}_i y_i}{\sum_{i=1}^{n} y_i^2}\hat{y}_i\right|$$

The mass normalization factor has the effect of ensuring that the overall asteroid mass, i.e. the integral over the volume V of the density predicted by the geodesyNet, is set as to minimize the resulting mean squared error. This adds one free parameter to the model, but a parameter that is analytically found at each step from the batch data. This way, the error backpropagation can focus on learning the deviation from a homogeneously filled volume V, and not, concurrently, find the absolute value of the body mass. In this work, we experimented with a number of different losses (see Table S5) with the κMAE being the final choice for the best performing models.

Supplementary Method 2

To compute the acceleration $\mathbf{a}(\mathbf{r})$ induced by some density distribution $\rho(\mathbf{x})$ (e.g. from the last layer of the geodesyNet) we use the formula:

$$\mathbf{a}(\mathbf{r}) = G\int_{\mathbf{x}\in V}\frac{\rho(\mathbf{x})}{|\mathbf{r}-\mathbf{x}|^3}(\mathbf{r}-\mathbf{x})\,dV. \tag{1}$$

where G is the Cavendish constant. Due to the lack of analytical solutions, these integrals have to be computed numerically in such a way as to allow for the error to be backpropagated through the integral. Given that the integral for the acceleration is a triple integral, getting a sufficiently accurate approximation is the computationally most expensive factor during the network training. For the computation of the integral approximation the network has to be evaluated at a large number of points (in our case 3 to $5 \cdot 10^5$ points). At the time of the experiments no public code was available to compute more than one-dimensional integrals while maintaining automatic differentiation capabilities on a GPU. Hence, we implemented two methods for this problem ourselves. We tested two methods for solving the necessary integral numerically, a

Monte Carlo Integrator (*1*) and a classical composite trapezoidal rule. Comparatively simple methods were chosen as a full vectorization and implementation in *PyTorch* was necessary for computational efficiency and to enable backpropagation through the integration. We showcase the convergence properties in the supplementary material in Figure S1. In practice, each training iteration of the network requires the evaluation of the integral for all sampled points r. However, the evaluation of the network $\rho(\mathbf{x})$ has to be performed only once per iteration. Thus, an efficient implementation of the numerical integration algorithm is paramount. With the chosen implementation, our integration methods demonstrated absolute errors with magnitude $\approx 1e-4$. Even though neural networks are somewhat robust to noise (such as numerical errors), this is likely a limiting factor for the network's obtainable accuracy and we suspect that improving the numerical accuracy of the integral computation may enable even better results.

Supplementary Method 3

To improve the resulting neural density fields for heterogeneous bodies we developed a separate training procedure relying on the knowledge on the body shape information. Without such information the neural density field, while still able to reproduce the observed gravity measurements within a good approximation, tends to create an homogeneous body contained in ∂V_B. This innate bias of networks towards low frequencies discussed by *Rahaman et al.* (*2*) is unavoidable as the loss function is not able to distinguish between the two possible and equally valid solutions – a consequence of the fact that the gravity inversion problem is ill-posed. In those cases where the body shape is known, though, the network can, and should, be informed on what areas of the volume V do contain a vanishing density and which ones don't. A simple, albeit elegant, solution to indirectly introduce this information in the loss is to train the geodesyNet to learn the difference between the measured acceleration y_{nu} and that created assuming a perfectly homogeneous body y_u – which we can compute since the body shape is

assumed as known.

In our experiments on differential training, consistently with the rest of our setup, we used mascon models to compute both y_u and y_{nu}, but it is worth mentioning here that in different setups, the computation of y_u could also be done using a polyhedral gravity approach which is an exact solution for a polyhedral shaped body. The geodesyNet is then tasked with predicting the local density difference between the bodies that causes the difference in acceleration. Thus, for each sampling point r_i, based on ground-truth acceleration y_u and y_{nu} for the homogeneous and heterogeneous, respectively, we optimize the network parameters θ to minimize

$$\mathcal{L}_{\kappa MAE} = \frac{1}{n}\sum_{i=1}^{n} \left| y_{nu} - y_u - \kappa \tilde{y} \right| . \tag{2}$$

Note that this implies the availability of a homogeneous density model of the target body, and hence knowledge of its shape. In practice, this may be obtainable even on a spacecraft as shown by Bandyonadhyay *et al.* (*3*). Then, the expected homogeneous acceleration y_u, would be precomputed from a polyhedral gravity model, for example, while y_{nu} is observed during flight.

Supplementary Method 4

In the case of the asteroids 433 Eros, 101955 Bennu and 25143 Itokawa and the comet 67P Churyumov–Gerasimenko, we obtain the high-fidelity polyhedral model shapes reconstructed from the various instruments on-board spacecraft that visited the asteroid: for Eros and Itokawa we use the models produced by *Robert Gaskell* (*4*) (*5*), for Bennu we use the model made available by the Osirix-REX team (*6*) and for Churyumov-Gerasimenko we use the model made available by the European Space Agency (*7*). Since the polyhedral models of the asteroid surface are not suitable to represent the gravitational field of heterogeneous bodies, we transform the surface meshes into mascon models first creating a constrained Delaunay tetrahedraliza-

tion (*8*) and then placing a mass m_j at the centroid of each resulting tetrahedron.

To further add variety to our dataset, we generate two additional mascon models not representative of any real body in the solar system: Planetesimal and Torus. The model for Planetesimal is obtained from N-Body simulations made at the Max Planck Institute for Astronomy using the SMC paradigm (*9*) and aimed at reproducing planetesimal formation. A final, statically stable planetesimal, is extracted from the simulation and used directly. To obtain Torus, instead, we create, similarly to what was done for the Solar System bodies, a mascon model from a starting polyhedral mesh representing a toroidal object. Both Planetesimal and Torus are then arbitrarily assumed to have the same mass and diameter as that of the comet Churyumov–Gerasimenko. For all bodies, we consider the homogeneous case by setting the values of the masses m_j to a value proportional to the corresponding tetrahedron volume and taking care that the sum of all masses reconstructs the actual body mass. For the planetesimal case we simply set all values as equal since the body is itself a stable aggregate of spherical masses.

For Bennu, Itokawa and Planetesimal we also generate models having a heterogeneous mass distribution. In the case of Bennu, flight data from the Osirix-REX mission indicated a possible area with a lower density at the equator (*10*), hence we generate the heterogeneous version of the model with a fictitious higher density area in the polar regions. We multiply the values of the mascon masses taken from the homogeneous model and belonging to the polar regions, by an arbitrary factor $f = 2.0$ and we renormalize the overall asteroid mass. Similarly, in the case of Itokawa, flight data revealed a possible higher density in the rubble pile head (*11*). We thus create a heterogeneous version of the mascon model by multiplying all mascons masses taken from the homogeneous model and belonging to the asteroid head by a factor $f = 1.6$, in agreement with the data published by Lowry et al. (*12*), and normalizing again the overall asteroid mass. In the case of the Planetesimal, we generate a hollow structure by setting all mascon masses belonging to an internal spherical region to zero, and renormalizing the remaining

mascon masses.

To standardize all our experiments and be able to develop a unique numerical pipeline for all the very different bodies here studied, we introduced non-dimensional units for the length, the mass and time. In particular, we set the integration volume V in Eq.(1) to be the hypercube $[-1, 1]^3$ and rescale the body mascon model so that the maximum absolute value of its coordinates is $\delta_{max} = 0.8$. Hence we derive the unit length L for the body. We then set the unit of mass to the body mass M and derive the value of the units of time by setting the Cavendish constant G to one. The resulting units as well as other parameters of the various models used for each body are shown in the supplementary materials Table S3.

Supplementary Method 5

In the work from *Wittick and Russell* (*13*) the authors propose an original technique to represent gravity fields of irregular bodies and study thoroughly its accuracy in the case of the asteroid Ero. Different choices are made for various free parameters of the approach. In their work both mascon and spherical harmonics methods are leveraged in the attempt to achieve maximum accuracy and validity of the proposed model. The resulting approach called MultiMESH leads to a combination of manual and automated efforts to build the final model. The availability of a shape model forms the basis of the technique allowing to optimally pack mascon masses below the asteroid surface and to choose a good trade-off with the number of harmonics employed. Their work is of interest here as it is the only work we could find in the literature reporting data in a form suitable to give some comparative information on the accuracy achieved by our geodesyNet approach.

In Table S4 we report numbers that are comparable directly to those reported by *Wittick and Russell*, in particular to their Table 3. It must be noted that there are still a few differences in the way the two set of results were formed and that affect the quantitative numbers reported. To start

with the actual position of the points sampled at the different altitude ranges differ. Secondly, the ground truth used is different as *Wittick and Russell* make use of a smoother polyhedral gravity model – not of a mascon model – which instead we use as to be able to also consider non homogeneous bodies. Clearly, at low altitudes, the mascon model creates a challenging gravitational environment to learn.

Overall, it is remarkable that a geodesyNet, without using any shape model and using a fully automated and generic pipeline, achieves performances – for the case of Eros – that are comparable, albeit often inferior, to some of those reported by *Wittick and Russell* in their work. It is here also worth noting that introducing the use of a shape model, and thus allowing for the differential training approach of a geodesyNet would here result in orders of magnitude smaller errors since the network would have to learn a vanishing neural density field representing the deviation of the ground truth – homogeneous – from a homogeneous body. It is then understood how the comparison made here, while informative, must be considered as extremely preliminary and that the creation of a shared dataset and the establishment of a common validation practice is necessary to firmly grasp on quantitative differences between possible approaches.

Supplementary Method 6

We here report the necessary formulas allowing to compute spherical harmonics models out of mascon models and geodesyNet models. We use the following formal definitions for the description of the gravitational potential (*14, 15*):

$$U(r,\theta,\phi) = \frac{\mu}{r}\sum_{l=0}^{l=\infty}\sum_{m=0}^{m=l}\left(\frac{r_0}{r}\right)P_{lm}(\cos\theta)\cdot(C_{lm}\cos m\phi + S_{lm}\sin m\phi)$$

where μ is the gravitational parameter of the main body, r_0 some characteristic length, P_{lm} the Legendre associated polynomials and C_{lm}, S_{lm} the Stokes coefficients. The Stokes coefficients

relate to the body density via the formulas:

$$C_{lm} = \frac{(2-\delta_{m,0})}{M}\frac{(l-m)!}{(l+m)!}\int_V \rho\left(\frac{r}{r_0}\right)^l \cdot P_{lm}(\cos\theta)\cos m\phi dV \tag{3}$$

and,

$$S_{lm} = \frac{(2-\delta_{m,0})}{M}\frac{(l-m)!}{(l+m)!}\int_V \rho\left(\frac{r}{r_0}\right)^l \cdot P_{lm}(\cos\theta)\sin m\phi dV \tag{4}$$

where the Dirac's delta $\delta_{i,j}$ has been used as well as the body total mass M. We also use the normalization factor N_{lm} defined as:

$$N_{lm} = \sqrt{\frac{(l+m)!}{(2-\delta_{m,0})(2l+1)(l-m)!}}$$

so that we make use of normalized Stokes coefficients defined by $\left\{\tilde{C}_{lm}, \tilde{S}_{lm}\right\} = \{C_{lm}, S_{lm}\} N_{lm}$. These normalized Stokes coefficients is what we will here call spherical harmonics. One can easily compute the spherical harmonics out of a GeodesyNet model by computing by numerical quadrature the formulas in Eq.(3, 4). In a similar fashion, one can also compute the spherical harmonics for a mascon model (and hence our ground truths), by using the formal expression containing Dirac's delta functions,

$$\rho = \sum_j m_j \delta(\mathbf{r} - \mathbf{r}_j)$$

for the density of a mascon model and plugging it in Eq.(3, 4). In Table S6 we report, in the case of 433 Eros, the values of the ten most significant spherical harmonics as computed from the geodesyNET model and compared to the ground truth values.

Supplementary Method 7

It is interesting to analyze the difference between a mascon model and a geodesyNet model to understand in depth their properties. To do so, let us compare the expression of the gravitational potential in a generic point $\mathbf{r}$ derived from a mascon model and from a geodesyNet. In the first

case we have:

$$U(\mathbf{r}) = -\sum_j \frac{m_j}{r_j}$$

where the sum is made over all positions occupied by mascons. For the geodesyNet, we have:

$$U(\mathbf{r}) = -\int_V \frac{\rho(\mathbf{r}_j)}{rj} dV = -\sum_j \frac{w_j \mathcal{N}(\mathbf{r}_j)}{rj}$$

where we have explicitly written the generic expression of a numerical quadrature (w_j are the quadrature weights). In this last expression the sum is extended to all the quadrature points and $\mathcal{N}$ denotes the density value as predicted on those points by a geodesyNet. Let us now assume that the masses of the mascon model are placed exactly on the same points as the ones used for the quadrature, as would for example be the case for a mascon cube and a quadrature formula using a uniform grid. By comparing the above two expressions it is clear that, for a geodesyNet to return the same result as those of the mascon model we must have:

$$w_j \mathcal{N}(\mathbf{r}_j) = m_j \tag{5}$$

In other words, in order to reproduce a mascon cube model prediction, the geodesyNet would need to reproduce the mascon values at each one of the integration points. This allows to understand how a geodesyNET with N parameters, when used in a quadrature scheme using M points, can be as good as a mascon cube model having M parameters, provided that it reproduces the mascon values as shown by Eq.(5). Clearly, this will be possible whenever a the spatial correlation existing between the different m_j values is exploited, in which case the geodesyNet will act similarly to a compression algorithm, packing M mascon values into N network parameters.

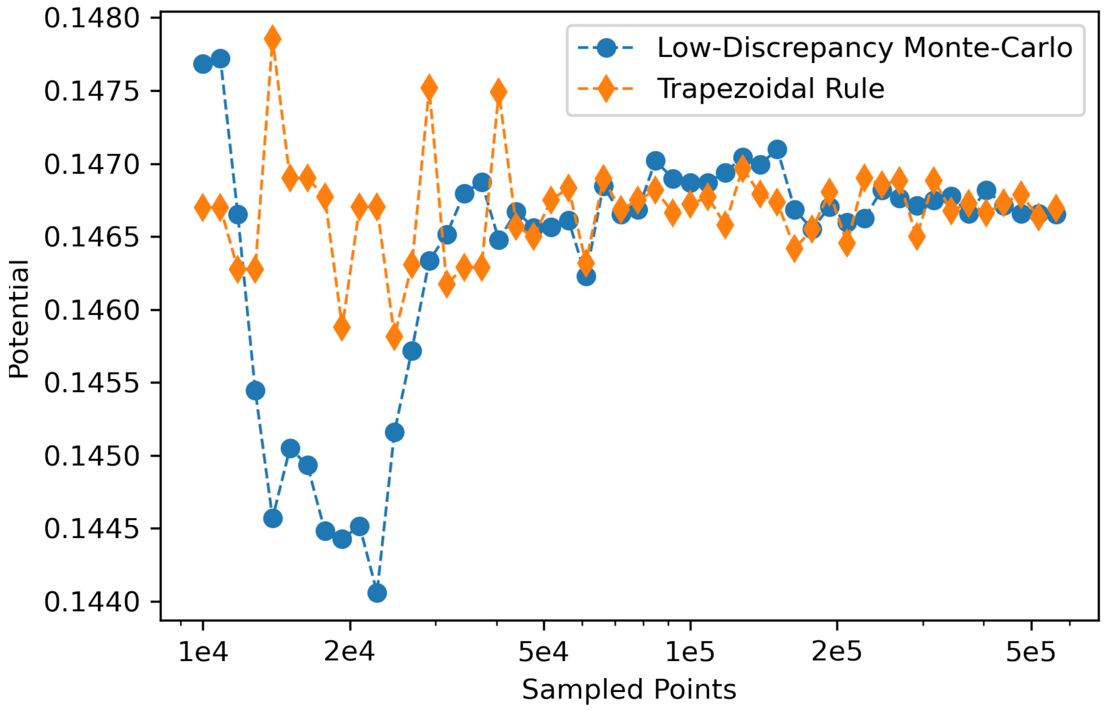

Figure S1: Convergence study of different tested integration methods on computing the potential at a point above Churyumov-Gerasimenko. A larger number of integration sample points is infeasible due to memory limitations. No analytic solution is available.

Table S1: Parameter study of the amount of hidden layers and neurons per layer on Churyumov–Gerasimenko.

		Sampling Altitudes			Absolute Errors			Relative Errors		
Hidden Layers	Neurons	$h_{low}[m]$	$h_{med}[m]$	$h_{hi}[m]$	$\epsilon_{low}[m/s^2]$	$\epsilon_{med}[m/s^2]$	$\epsilon_{hi}[m/s^2]$	$\epsilon_{low}[\%]$	$\epsilon_{med}[\%]$	$\epsilon_{hi}[\%]$
3	100	125	250	625	4.69E-07	1.34E-07	3.00E-08	0.781	0.251	0.083
5	100	125	250	625	2.28E-07	6.69E-08	1.70E-08	0.379	0.125	0.045
7	100	125	250	625	2.18E-07	7.71E-08	2.44E-08	0.362	0.145	0.065
9	100	125	250	625	3.33E-07	1.70E-07	5.56E-08	0.556	0.319	0.146
9	10	125	250	625	4.28E-06	2.05E-06	4.14E-07	7.092	3.750	1.044
9	25	125	250	625	1.04E-06	3.71E-07	9.21E-08	1.734	0.689	0.240
9	50	125	250	625	3.67E-07	1.26E-07	3.48E-08	0.606	0.236	0.092
9	75	125	250	625	4.39E-07	2.20E-07	8.54E-08	0.730	0.410	0.221
9	100	125	250	625	3.33E-07	1.70E-07	5.56E-08	0.556	0.319	0.146

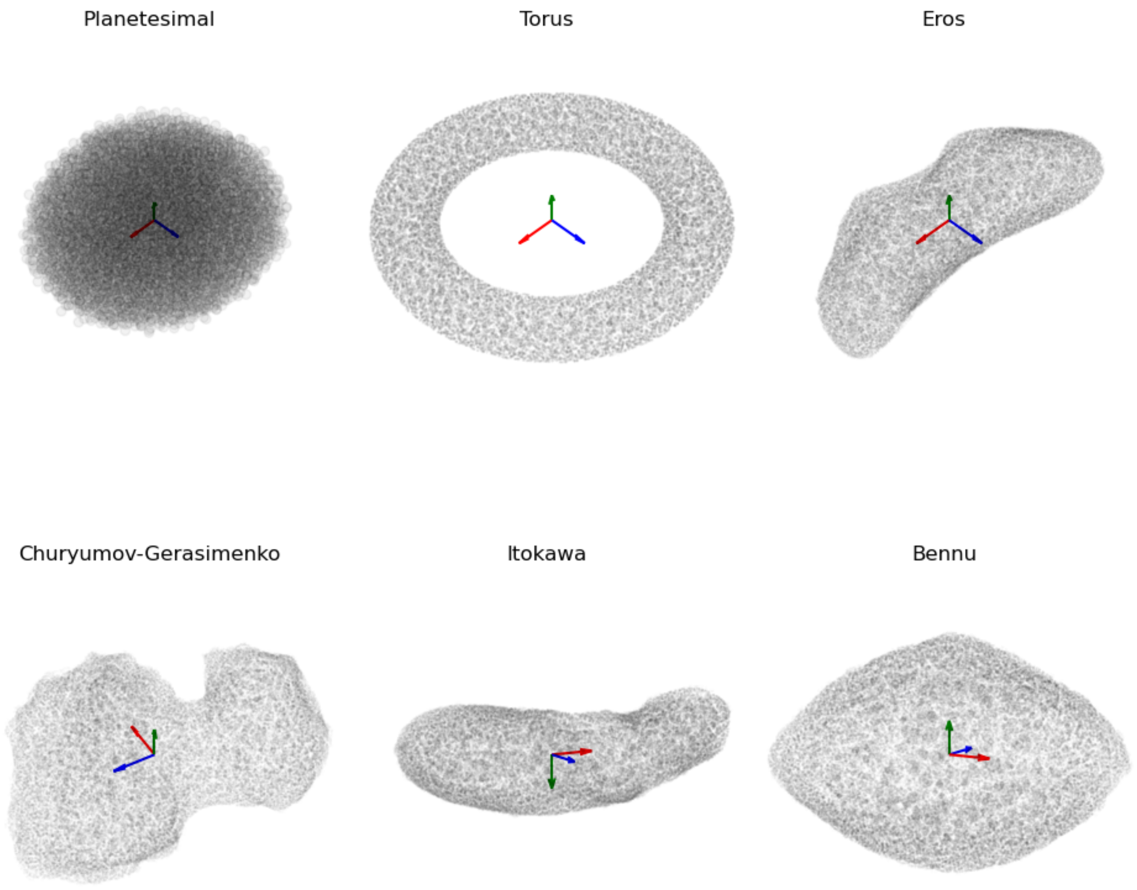

Figure S2: Mascon models for the six bodies considered. The dimension of each mascon is constant, hence the actual mass distribution inside the bodies is not visible, only their shape. Principal axes, used throughout this work, are also shown for convenience.

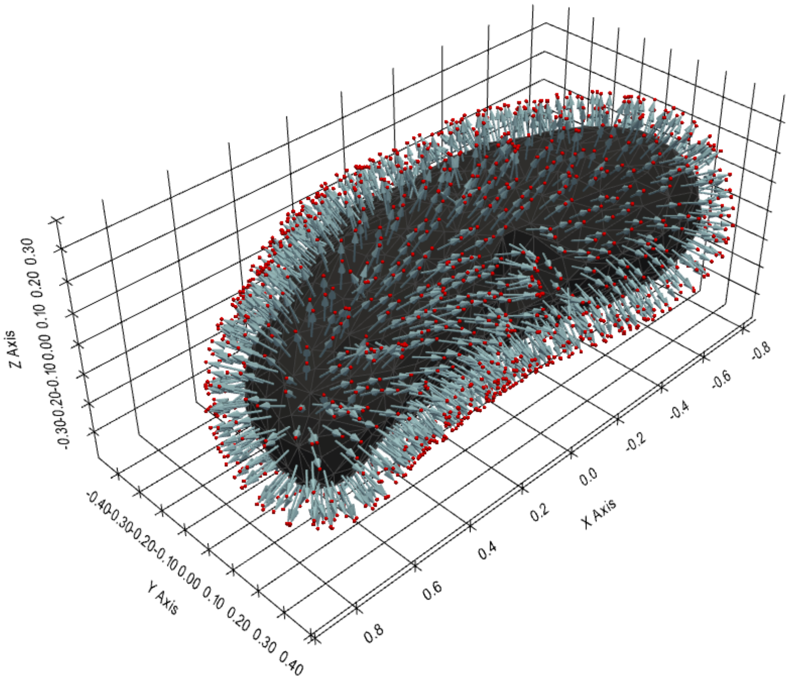

Figure S3: Visualization of the validation point sampling on the example of Eros. Points (red) are sampled at specific altitude - 0.1 in non-dimensional units, equivalent to 2042.5m - using the normal vectors (blue) of the body mesh triangles (grey). Points with mismatching altitude due to the non-convex nature of the mesh are automatically discarded. Additional details are given in the Support Information Text.

Table S2: Parameter study of the ω parameter in the Siren network without differential training on Churyumov–Gerasimenko and Eros. Depending on altitude optimal results obtained with $\omega = 15$ or $\omega = 30$ for higher and lower altitude, respectively.

		Sampling Altitudes			Absolute Errors			Relative Errors		
Body	ω	$h_{low}[m]$	$h_{med}[m]$	$h_{hi}[m]$	$\epsilon_{low}[m/s^2]$	$\epsilon_{med}[m/s^2]$	$\epsilon_{hi}[m/s^2]$	$\epsilon_{low}[\%]$	$\epsilon_{med}[\%]$	$\epsilon_{hi}[\%]$
Churyumov-Gerasimenko	1.0	125	250	625	3.85E-06	2.94E-06	4.61E-07	6.473	5.527	1.221
Churyumov-Gerasimenko	15	125	250	625	3.91E-07	1.28E-07	3.91E-08	0.649	0.239	0.102
Churyumov-Gerasimenko	30	125	250	625	3.33E-07	1.70E-07	5.56E-08	0.556	0.319	0.146
Churyumov-Gerasimenko	45	125	250	625	5.13E-07	2.91E-07	1.23E-07	0.854	0.544	0.321
Churyumov-Gerasimenko	60	125	250	625	1.38E-06	6.49E-07	3.76E-07	2.276	1.208	0.991
Churyumov-Gerasimenko	75	125	250	625	1.86E-06	9.24E-07	3.32E-07	3.088	1.728	0.879
Eros	1.0	817	1630	4080	1.24E-04	6.41E-05	1.60E-05	8.519	5.079	2.028
Eros	15	817	1630	4080	6.62E-06	1.92E-06	4.21E-07	0.451	0.155	0.052
Eros	30	817	1630	4080	6.82E-06	3.22E-06	9.96E-07	0.467	0.259	0.123
Eros	45	817	1630	4080	1.02E-05	4.93E-06	1.48E-06	0.698	0.395	0.182
Eros	60	817	1630	4080	2.88E-05	1.46E-05	5.24E-06	1.975	1.178	0.645
Eros	75	817	1630	4080	1.88E-05	1.07E-05	4.33E-06	1.335	0.894	0.557

Table S3: Overview of various parameters associated to the ground-truth bodies. The number of vertices of the shape model used, as well as of its low-precision version is reported. The low-precision version of the shape model is used to determine efficiently whether a point lies inside or outside the body. The body diameter, as well as the mass and length units used is also shown.

Body	# of vertices	# of vertices (low-poly)	# of mascons	Diameter (km)	Mass unit (kg)	Length unit (km)
Bennu	7374	739	37799	0.563	7.329e10	0.352
Churyumov-Gerasimenko	9149	916	43599	5.002	9.982e12	3.126
Eros	7374	739	39554	32.66	6.687e15	20.413
Itokawa	8112	813	41748	0.561	3.51e10	0.350
Planetesimal	6534	6534	4922	5.002	9.982e12	3.126
Torus	4044	404	20898	5.002	9.982e12	3.126

Table S4: Parameter study of model size and obtained root mean square error (RMS) on Eros. Results indicate comparable performance with prior results by Wittick & Russell (*13*). Sampling altitudes were 817m, 1633m and 4083m for low, medium and high altitude, respectively. Results used $\omega = 15$ for the Siren network.

Configuration			Absolute RMS Errors			Relative RMS Errors		
Hidden Layers	Neurons	Parameters	$\epsilon_{low}[m/s^2]$	$\epsilon_{med}[m/s^2]$	$\epsilon_{hi}[m/s^2]$	$\epsilon_{low}[\%]$	$\epsilon_{med}[\%]$	$\epsilon_{hi}[\%]$
5	10	601	2.55E-04	9.17E-05	1.43E-05	5.578	2.376	0.587
5	25	3376	1.10E-04	4.31E-05	4.53E-06	2.390	1.057	0.184
5	50	13001	6.53E-05	2.40E-05	2.19E-06	1.424	0.589	0.091
5	75	28876	5.10E-05	1.69E-05	2.50E-06	1.133	0.429	0.125
5	100	51001	4.33E-05	1.28E-05	1.68E-06	0.963	0.328	0.078
9	100	91401	2.53E-05	6.88E-06	1.40E-06	0.571	0.184	0.060

Table S5: Parameter study of loss, encoding and network architecture on Churyumov–Gerasimenko.

Configuration			Sampling Altitudes			Absolute Errors			Relative Errors		
Model	Loss	Encoding	$h_{low}[m]$	$h_{med}[m]$	$h_{hi}[m]$	$\epsilon_{low}[m/s^2]$	$\epsilon_{med}[m/s^2]$	$\epsilon_{hi}[m/s^2]$	$\epsilon_{low}[\%]$	$\epsilon_{med}[\%]$	$\epsilon_{hi}[\%]$
Siren	κMAE	Spherical	125	250	625	6.14E-07	3.48E-07	1.40E-07	1.026	0.648	0.358
Siren	κMAE	Directional	125	250	625	4.88E-07	2.26E-07	6.93E-08	0.814	0.419	0.175
Siren	κMAE	Direct	125	250	625	3.33E-07	1.70E-07	5.56E-08	0.556	0.319	0.146
Siren	MSE	Direct	125	250	625	1.04E-06	4.15E-07	1.50E-07	1.722	0.771	0.398
Siren	Root κMAE	Direct	125	250	625	7.48E-07	3.38E-07	1.00E-07	1.233	0.627	0.258
Siren	κMSE	Direct	125	250	625	1.28E-06	5.59E-07	2.33E-07	2.113	1.041	0.618
NeRF	κMAE	Direct	125	250	625	1.35E-06	5.32E-07	5.73E-08	2.261	0.994	0.149

Table S6: The eight most significant spherical harmonics for 433 Eros as computed from the geodesyNET and the ground truth.

	C_{22}	C_{20}	C_{44}	C_{42}	C_{40}	S_{33}	S_{55}	S_{77}
ground truth	8.016e-02	-4.829e-02	1.631e-02	-1.517e-02	1.091e-02	-1.377e-02	-9.266e-03	-5.136e-03
geodesyNET	8.015e-02	-4.827e-02	1.620e-02	-1.514e-02	1.077e-02	-1.371e-02	-9.216e-03	-5.047e-03

Supplementary References

1. R. E. Caflisch, *et al.*, Monte carlo and quasi-monte carlo methods. *Acta numerica* **1998**, 1–49 (1998).

2. N. Rahaman, A. Baratin, D. Arpit, F. Draxler, M. Lin, F. Hamprecht, Y. Bengio, A. Courville, *International Conference on Machine Learning* (PMLR, 2019), pp. 5301–5310.

3. S. Bandyonadhyay, I. Nesnas, S. Bhaskaran, B. Hockman, B. Morrell, *2019 IEEE Aerospace Conference* (IEEE, 2019), pp. 1–13.

4. R. Gaskell, Eros polyhedral model (2008). (available at `https://sbn.psi.edu/pds/resource/erosshape.html`).

5. R. Gaskell, Itokawa polyhedral model (2007). (available at `https://darts.isas.jaxa.jp/planet/project/hayabusa/shape.pl`).

6. NASA/Goddard/University of Arizona, Bennu polyhedral model (2020). (available at `https://www.asteroidmission.org/updated-bennu-shape-model-3d-files`).

7. ESA/Rosetta/MPS, Churyumov-Gerasimenko polyhedral model (2014). (available at `https://sci.esa.int/web/rosetta/-/54728-shape-model-of-comet-67p`).

8. H. Si, TetGen, a Delaunay-based quality tetrahedral mesh generator. *ACM Transactions on Mathematical Software (TOMS)* **41**, 1–36 (2015).

9. J. Fleischmann, R. Serban, D. Negrut, P. Jayakumar, On the Importance of Displacement History in Soft-Body Contact Models. *Journal of Computational and Nonlinear Dynamics* **11** (2016).

10. D. Scheeres, A. French, P. Tricarico, S. Chesley, Y. Takahashi, D. Farnocchia, J. McMahon, D. Brack, A. Davis, R.-L. Ballouz, *et al.*, Heterogeneous mass distribution of the rubble-pile asteroid (101955) Bennu. *Science advances* **6**, eabc3350 (2020).

11. A. Fujiwara, J. Kawaguchi, D. Yeomans, M. Abe, T. Mukai, T. Okada, J. Saito, H. Yano, M. Yoshikawa, D. Scheeres, *et al.*, The rubble-pile asteroid Itokawa as observed by Hayabusa. *Science* **312**, 1330–1334 (2006).

12. S. Lowry, P. Weissman, S. Duddy, B. Rozitis, A. Fitzsimmons, S. Green, M. Hicks, C. Snodgrass, S. Wolters, S. Chesley, *et al.*, The internal structure of asteroid (25143) Itokawa as revealed by detection of YORP spin-up. *Astronomy & Astrophysics* **562**, A48 (2014).

13. P. T. Wittick, R. P. Russell, Mixed-model gravity representations for small celestial bodies using mascons and spherical harmonics. *Celestial Mechanics and Dynamical Astronomy* **131**, 31 (2019).

14. P. Tricarico, Global gravity inversion of bodies with arbitrary shape. *Geophysical Journal International* **195**, 260–275 (2013).

15. C. F. Yoder, Astrometric and geodetic properties of earth and the solar system. *Global Earth Physics: A Handbook of Physical Constants* **1**, 1 (1995).
